# Neuromuscular Stimulation of the Common Peroneal Nerve: Improved Pain Trajectory in Chronic Venous Leg Ulcers

**DOI:** 10.3390/jcm15135234

**Published:** 2026-07-04

**Authors:** Karen Staines, Samantha Holloway, Duncan Bain, Keith Harding

**Affiliations:** 1 Accelerate CIC, Centenary Wing, St Joseph’s Hospice, Mare St, London E8 4SA, UK; 2 Centre for Medical Education, College of Biomedical and Life Sciences, Cardiff University School of Medicine, 9th Floor, Heath Park, Cardiff CF14 4YS, UK; hollowaysl1@cardiff.ac.uk; 3 Duncan Bain Consulting, 22 Gypsy Lane, Kings Langley WD4 8PR, UK; 4 School of Medicine, UHW Main Building, Cardiff University, Heath Park, Cardiff CF14 4XN, UK

**Keywords:** venous leg ulcers, VAS pain score, self-controlled, pain, neuromuscular electrostimulation

## Abstract

**Background**: Pain is among the most problematic aspects of chronic wounds. It adversely affects not only physical function but also social and psychological aspects of life. In concert, these effects can seriously impinge on quality of life. Despite affecting up to 80% of patients with leg ulcers, pain is reported to be under-treated. **Methods**: A secondary, within-subject controlled study design comparing wound pain intensity using a visual analogue scale of venous leg ulcer patients over 4 weeks receiving different interventions. In total, 29 patients received multi-layer compression over 4 weeks, followed by neuromuscular stimulation (NMES) of the leg muscle pump in addition to compression for a further 4 weeks. Paired comparison was then made of the pain gradient (rate of change in pain) between the two phases. A second cohort of 22 patients received only multi-layer compression throughout both 4-week phases. The trial was reported following the CONSORT guidelines. **Results**: Introduction of NMES at week 4 was accompanied by a significant increase in the rate of pain reduction over the following 4 weeks. Patients receiving standard care for the second 4-week phase experienced no change in the pain trajectory. **Conclusions**: 1 Hz NMES of the common peroneal nerve to activate the leg muscle pump is accompanied by an improved pain trajectory in patients with chronic venous leg ulcers. This mirrors the improvement in the rate of reduction in wound size previously reported in the same cohort. Pain is a common feature of chronic wounds and mitigation of pain is likely to have a profound impact on the quality of life of individuals with chronic venous leg ulcers.

## 1. Introduction

Patients frequently report pain as being the worst aspect of chronic wounds [1], even when taken alongside other serious medical problems [2]. It adversely affects not only physical function, but also social and psychological aspects of life [3]. In concert, these effects can seriously impinge on quality of life [4,5]. However, despite affecting up to 80% of patients with leg ulcers [6], pain is reported to be under-treated [7]. Treatment of pain consists predominantly of analgesics [8], and has been reported to be inadequate [9]. The Chronicity of leg ulcers has also been linked to the chronicity of pain, often leading to neuropathy [10].

A recently conducted self-controlled study [11] demonstrated that the Neuromuscular Electrostimulation (NMES) geko^®^ device (Firstkind Ltd., Daresbury, UK) accelerated the healing of venous leg ulcers in 29 patients. The primary endpoint of that study was the rate of reduction in wound size over a 4-week treatment period, with each patient self-controlled to a 4-week run-in period. The study demonstrated that the introduction of the intervention was accompanied by an increased rate of wound size reduction, and these findings have been published elsewhere [12]. In the self-controlled study design, each subject’s own rate of wound size reduction during a run-in phase was compared with that of the same subject during the treatment phase. This eliminates much of the inter-group heterogeneity and many of the confounders inherent in wound studies, thereby greatly improving the statistical sensitivity and power of the study [13]. While the primary endpoint of that study was the rate of advance of the wound edge, secondary endpoints were collected for descriptive reporting, including adherence, infection rates, percentage complete healing, quality of life scores EQ-5D-5L, Cardiff Wound Impact Schedule (CWIS), Venous Clinical Severity Score (VCSS), and Visual Analog Score (VAS) for pain. Similar to wound size, VAS was measured weekly over a 4-week run-in period, followed by a 4-week treatment period. This allows the rate of reduction in VAS to be compared between run-in and intervention periods, with each patient acting as his/her own control. This paper reports on the data related to pain in terms of change to the pain gradient for the first time. The paper also puts forward some suggested hypotheses for how the NMES device may mitigate the experience of pain in individuals with venous leg ulcers.

## 2. Methods

A total of 60 subjects with venous leg ulcers were randomised to 2 groups. One received standard of care (SOC), consisting of multi-layer multi-component compression bandaging for the entire 8-week trajectory. The other group received SOC for the first phase of 4 weeks, followed by NMES for 12 h per day in addition to SOC for the second phase of 4 weeks. 

Participants were allocated to treatment groups using adaptive covariate randomization using an online randomization tool, applied centrally across the sites. Blinding of subjects was not possible, since it is obvious when a stimulus is applied to the leg. Assessors measuring the VAS marks were blind to the patient group. 

The study was registered and the protocol uploaded with clinicaltrials.gov: Registration: NCT03396731 (https://clinicaltrials.gov/search?term=NCT03396731&viewType=Card, accessed on 27 May 2026), first registered 10 January 2018. The investigations were conducted in accordance with the principles outlined in the Declaration of Helsinki (1975, revised in 2013), and were approved by the London Riverside Research Ethics Committee (REC reference 18/LO/0219, approved 30 January 2018. Data collection took place between 20 February 2020 and 21 September 2023.

**Figure 1:**
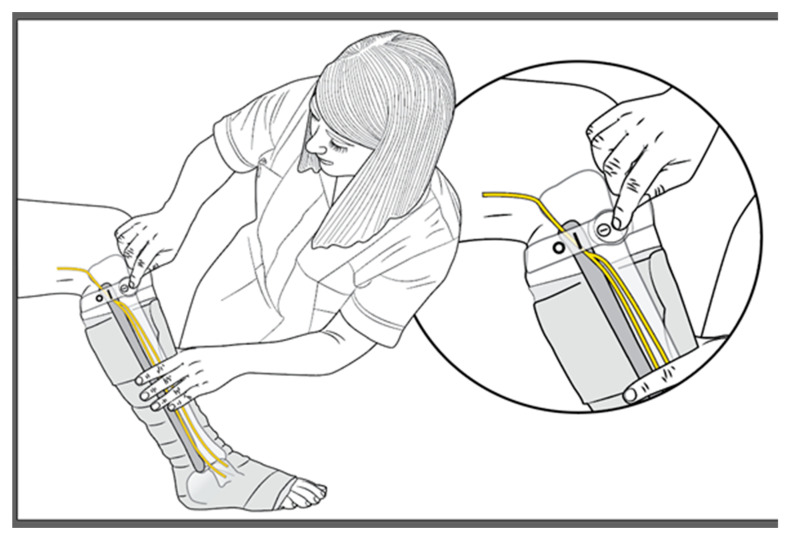
Positioning of NMES device over peroneal nerve for subject wearing lower-limb multi-layer compression therapy.

NMES consisted of the geko^®^ device (Firstkind Ltd., Daresbury, UK) applied superficially to the lateral aspect of the leg below the knee as per the manufacturer’s instructions. Figure 1 shows the positioning of the NMES device over the common peroneal nerve for a subject wearing lower-limb multi-layer compression therapy. The device intermittently activates the venous muscle pump, increasing venous, arterial, and microvascular perfusion [14]. The device settings were adjusted so that a perceptible intermittent twitch of the foot was visible. The device was used for 12 h treatment per day, and was removed and stored overnight between 12 h wear sessions.

### 2.1. Inclusion Criteria

- Aged 18 years or over and able to provide written informed consent.
- Chronic venous leg ulcer determined to be due to underlying venous disease following evaluation in a multidisciplinary clinic setting or by a vascular surgeon, GP, or Nurse Specialist.
- Ulcer size between 3 cm^2^ and 39 cm^2^ at study enrolment.
- Ulcer present for at least 6 weeks but no more than 5 years prior to study entry.
- Ankle-Brachial Pressure Index (ABPI) of 0.8–1.2 at study entry or within 8 weeks of study entry.
- No clinical infection in the study leg for a minimum of 48 h prior to study entry.
- No systemic antimicrobial treatment for a minimum of seven days prior to study entry was prescribed for the index ulcer wound infection.

### 2.2. Exclusion Criteria

- Known allergy to any of the protocol-stipulated treatments, or non-tolerance of multilayer, multicomponent compression therapy intended for the treatment of venous leg ulcers.
- History of significant haematological disorders (e.g., Sickle Cell disease).
- History of Deep Vein Thrombosis (DVT) within six months preceding study entry
- History of Pyoderma Gangrenosum or other inflammatory ulceration.
- Pregnancy or breastfeeding.
- Use of an investigational drug or device within four weeks prior to study entry that may interfere with this study.
- Use of any neuromodulation device.
- Surgery during the three months prior to study entry (such as abdominal, gynaecological, hip, or knee replacement).
- Any medication deemed by the Investigator to potentially interfere with the study treatment (e.g., systemic steroids).
- Participation in any other clinical study.

A total of 26 subjects were allocated to receive SOC, and 34 subjects to receive SOC plus NMES. In the SOC arm, one subject was excluded prior to intervention because of infection, two because wounds were too small (outside inclusion criteria) at randomization, and one owing to inflammation at the wound site. In the SOC plus NMES arm, one subject was excluded because of non-adherence to therapy, and three because wounds were too small at randomization. In total, 22 subjects completed the SOC arm, and 29 the SOC plus NMES arm.

**Figure 2:**
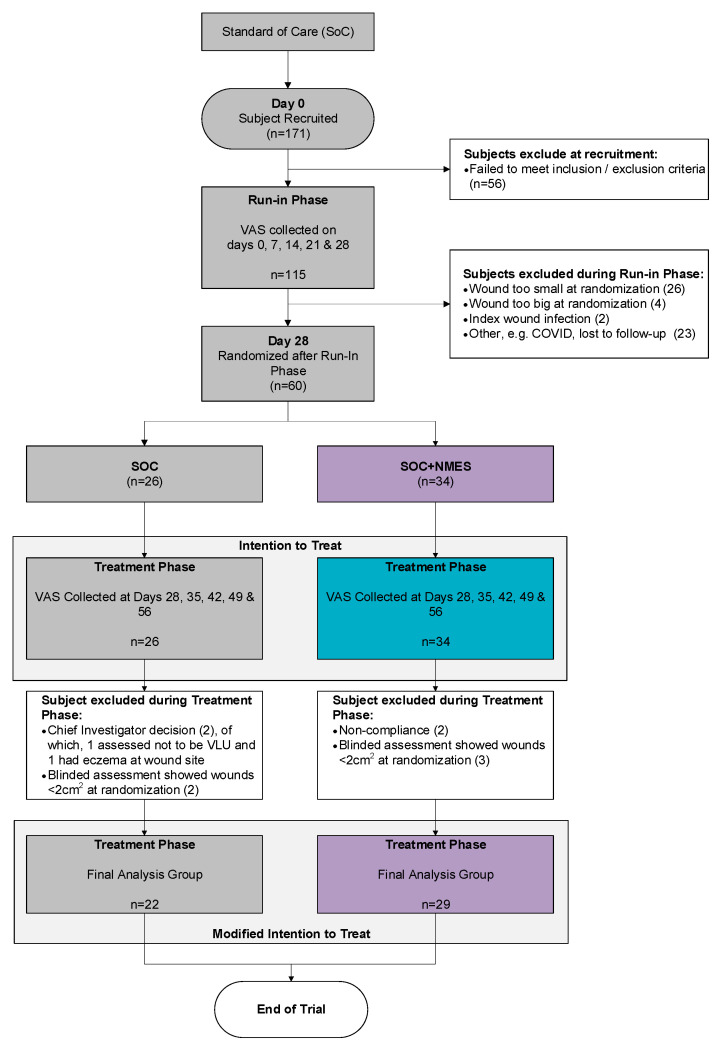
Study design and subject accountability.

Figure 2 presents the study flow and participant allocation. In both arms, subjects spent 4 weeks on a run-in control phase receiving SOC only, followed by a 4-week treatment phase. In the treatment phase, the SOC randomised group continued to receive SOC for a further 4 weeks, whereas the NMES randomised group received NMES in addition to SOC for a further 4 weeks. The study was not designed or powered to compare trajectories between the two randomised groups, but rather for each group to compare trajectories between the intervention phase and run-in phase, controlled within-patient. The primary endpoint of healing rate (rate of reduction in wound size) is reported elsewhere, and this paper concerns the trajectory of pain, measured as a secondary endpoint.

Subjects were asked to record their study target ulcer wound pain intensity using a 100 mm visual analogue scale (VAS). Subjects indicated their level of ulcer pain during the current week by placing a mark on a line 100 mm long, with 0 mm representing no pain, and 100 mm representing the worst possible pain [15,16]. VAS was recorded on day 0 and at every weekly visit until day 56 to give a weekly value. Linear regression was used to calculate a gradient (mm/day) representing the daily change in VAS during the 4-week run-in phase, followed by the 4-week treatment phase. VAS assessments were conducted by the same healthcare professional (tissue viability nurse), who would conduct them during each follow-up in the clinic. Patents were at rest and recumbent during this assessment. Data were collected using the Castor EDC platform.

## 3. Demographics

Table 1 presents the demographic breakdown of the subjects. Patients were randomised to compression only (SOC) or compression plus NMES for 12 h per day (SOC + NMES). No significant differences (Student’s *t*-test) were found between groups in terms of any of the parameters collected. It is not unexpected to see some inter-group variance in some parameters, e.g., ulcer age, considering the immense heterogeneity of venous ulcers. This level of heterogeneity would be problematic in a classic inter-cohort randomised controlled trial (RCT) design. However, this within-patient controlled design accommodates these inter-patient differences by comparing each subject’s intervention phase with his/her own run-in phase. Subject data was collected using the Castor EDC platform. The study is reported according to the CONSORT guidelines [17].

## 4. Results

**Figure 3:**
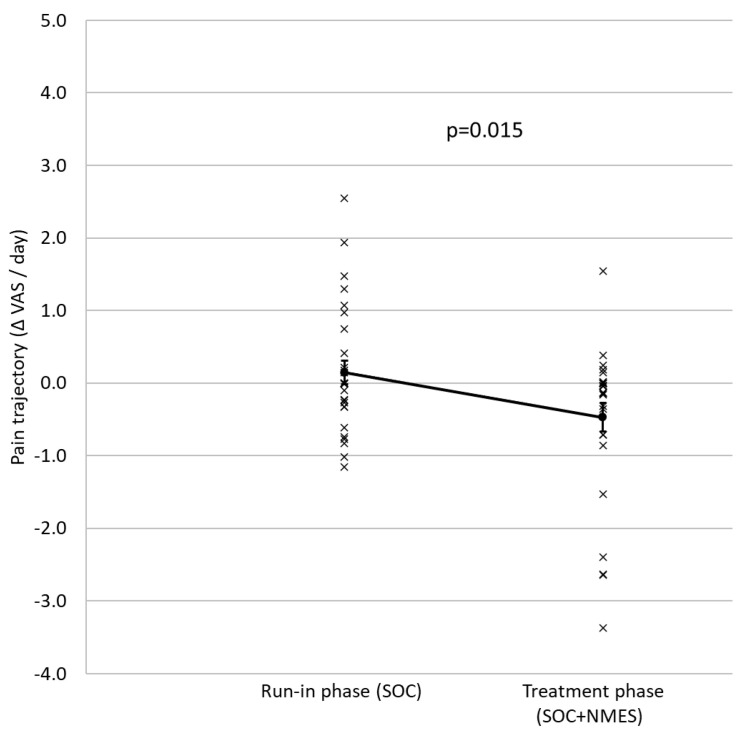
Pain trajectory during efficacy study. The solid line shows the change in mean trajectory from run-in phase to treatment phase, error bars show ±1 SE of the mean.

A value of pain gradient [18] for each phase for each subject is calculated by performing a linear regression on the 5-weekly time-point VAS values within that phase (week 0, 1, 2, 3, 4). These gradient values for all 29 subjects in the intervention group are shown in Figure 3, with the dark line showing the difference in the median gradients between the run-in (control) and treatment phase. During run-in, the gradient is close to zero, indicating no improvement in pain over the 4-week period. However, when the geko^®^ is introduced at day 28, there is a statistically significant improvement in pain trajectory (paired *t*-test, *p* = 0.015).

**Figure 4:**
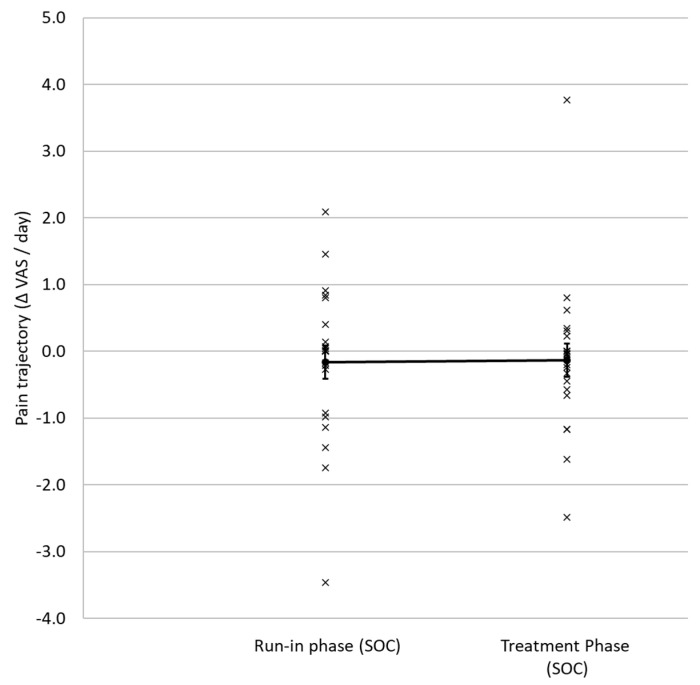
Pain trajectory in SOC-only arm. The solid line shows change in mean trajectory from run-in phase to treatment phase, error bars show ±1 SE of the mean.

This is in contrast to the control arm of the study (Figure 4), where patients received SOC only during both the 4-week run-in phase and the 4-week intervention phase. Here, no improvement in gradient is observed between the two phases of the study (paired *t*-test *p* = 0.946).

**Figure 5:**
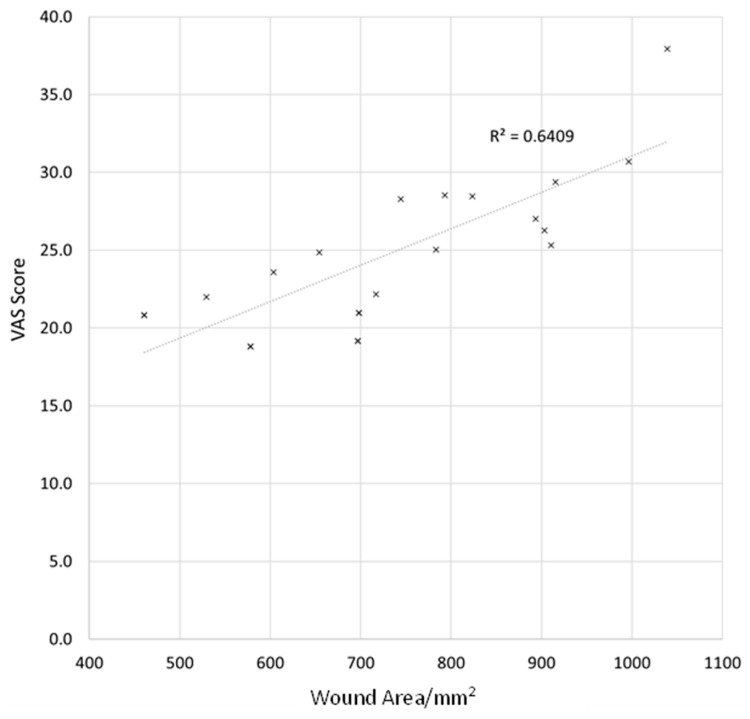
Correlation between wound size and pain.

An exploratory analysis of the correlation between wound area and pain is shown in Figure 5. Aggregating the data for both cohorts and plotting VAS against wound area gives the scatter plot shown. A correlation (R^2^ = 0.64) can be seen between VAS and wound area. Although this correlation is substantial, care must be taken to avoid the risk of temporal autocorrelation.

In the original primary endpoint study previously published [11,12], it is worth noting that the mean percentage area reduction (PAR) in wounds over 4 weeks with NMES was 43%, versus 17% in wounds without NMES (*t*-test, *p* = 0.01). This (more than twofold) increase in the rate of wound area reduction mirrors the increase in the rate of improvement of VAS when NMES is introduced, seen in Figure 2 and Figure 3. This further supports the notion that pain is related to ulcer size.

## 5. Discussion

In this trial, the intervention phase is compared to the run-in phase for each cohort, thus eliminating the substantial heterogeneity of wounds and wound patients. In this sense, the control group (receiving SOC only throughout both run-in and treatment phases) is not to be regarded as a comparator for the NMES group (receiving NMES in addition to SOC during the treatment phase). This separate control group serves purely to demonstrate the extent to which the baseline pain trajectory is stable over the 8-week period in the absence of any change in intervention: an assumption underpinning the validity of the self-controlled model. 

Pain in leg ulcers is multi-dimensional. According to one conceptual model [19], pain can be classified as *background*, *incident*, *procedural*, and *operative*. Background pain is caused by the underlying pathology of the leg ulceration and the wound itself; incident pain occurs during daily activities such as walking; procedural pain is caused by treatment (e.g., compression [20]); and operative pain is caused by debridement [21]. 

Equally, ulcer pain can be discriminated as *nociceptive* or *neuropathic*, or as *ischaemic* or *inflammatory* [22]. Nociceptive pain is caused by the activation of pain receptors local to the wound by tissue damage. Neuropathic pain is caused by a malfunction of the neural system communicating pain to the brain. Either of these, in turn, may be ischaemic in nature (whereby vessels fail to deliver nutrients or evacuate metabolites/lactate), or inflammatory (whereby engorgement/oedema activates receptors, or inflammatory mediators reduce thresholds for sensation) [23].

Further, the trajectory of pain can be plotted chronologically. Three distinct phases have been described [24]. In phase 1, leg ulcer pain is predominantly nociceptive; if this is allowed to persist (i.e., the ulcer does not heal), progression to phase 2 is marked by both nociceptive and neuropathic properties. Finally, phase 3 is refractory, long-term habitual pain. Equally, as an ulcer heals, pain improves dramatically according to the healing stage of the ulcer [25].

Ulcer pain may be further distinguished by ulcer aetiology [26]. In ulcers caused by venous insufficiency, blood ***leaving*** the leg is reduced, causing ischaemia. Accordingly, deep “aching” pain will often be associated with oedema and is alleviated by activity [27] (pumping blood out) and an elevated leg (so gravity helps blood out of the leg). This is contrasted with arterial aetiology, where the blood ***entering*** the leg is restricted [28]. Here, the increased metabolic demands of activity increase ischaemia and exacerbate acute pain (claudication), and pain is often relieved by a dependent leg (so gravity helps blood into the leg) [29].

It is acknowledged that using a single one-dimensional VAS tool may have limitations; however, the aim of the assessment was not to characterise, but to measure pain intensity. The quantitative approach of VAS allows us to examine the trajectory of total pain (regardless of its origin or nature), and so determine if an intervention alters that trajectory. VAS is the most commonly used tool to measure pain in clinical studies and is considered to be the gold standard for pain intensity measurements [30]. The European Wound Management Association (EWMA) recommends the use of unidimensional pain measurement tools [31], including VAS or the Numerical Rating Scale (NRS), which can be appropriate owing to ease of administration, validated reliability, sensitivity, and conceptual simplicity [32]. However, it is recognised that multi-dimensional tools such as the McGill Pain Questionnaire take into consideration the psychological and social factors that can impact on an individual’s QoL, so may provide a more comprehensive approach to the assessment of pain [32]. 

Intermittent 1 Hz Neuromuscular Electrostimulation (NMES) of the common peroneal nerve [33] is an intervention which has been shown to augment blood flow in the leg, by activating the muscles which operate the venous muscle pump. Increases to venous, arterial, and microcirculatory flow have been observed in healthy subjects as well as subjects with venous, arterial, and mixed pathologies [34,35]. This modality of NMEs differs from TENS (transcutaneous electrical nerve stimulation) used for pain, insofar as the latter does not elicit muscle twitches, but targets sensory receptors. 

In the study reported here, the introduction of NMES is seen to coincide with a significant and immediate improvement in the trajectory of pain in venous leg ulcers, measured weekly with VAS. The gradient of the mean pain trajectory post-introduction of NMES represents a small daily change in the mean VAS (approximately 0.5 mm/day), which would result in a 14 mm change in VAS over a 4-week period. This trajectory may be considered clinically important in the context of the minimum clinically important difference (MCID), which is variously determined in the range 5 mm–18 mm [36,37,38,39]. This is to be contrasted with the trajectory prior to intervention, where no improvement in pain was measured.

Several limitations must be considered. Foremost among these is the subjective nature of the metric for pain, and its susceptibility to inter-subject variances, though these are in part accommodated by the self-controlled model. It must be noted also that pain assessment was a secondary endpoint in a study designed primarily to measure wound healing, and which has been previously reported. Pain reduction would be more compellingly evaluated as a primary endpoint in a subsequent study. In addition, the impossibility of blinding subjects to the intervention means that a placebo effect cannot be ruled out. Data were not available on analgesic use over the 8-week period of this study, and it has not been possible to explore any dynamic interactions between interventions, pain, and reductions in analgesic use. 

Among the assumptions of inter-group and intra-group homogeneity is the supposition that compression was uniformly applied throughout the 8 weeks of the study. However, it was not possible to verify this, as no weekly interface pressure measurements were performed. A suggestion for future studies is to measure the interface pressure between skin and compression, as sensor technologies emerge to make this possible.

Questions exist as to external validity and the generalizability of the findings within this self-controlled model to a larger population. However, all these limitations plainly also apply to the traditional complete healing RCT model, combined with additional problems resulting from intergroup variances. 

Nevertheless, the finding of reduction in pain with NMES concurs with observations made in case series previously reported [40,41]. The current study was not intended to establish the mechanism for this effect, but there are several plausible hypotheses which relate to three main areas: ischaemia, reduction in ulcer size, and neuromodulation.

### 5.1. Ischaemia

Ischaemia is a causative factor of both nociceptive [42] and neuropathic [43] ulcer pain. It follows that an intervention which reduces ischaemia should have a beneficial effect on pain. Several published studies present mechanistic evidence that the NMES device improves arterial, venous, and microvascular blood supply in patients with ulcers of venous, arterial, diabetic, and mixed aetiologies [34,35,44]. This mechanism would fit with the shape of the curve seen in Figure 2, where most of the benefit is observed immediately when NMES is introduced. The improvements to the haemodynamic parameters when using NMES are seen acutely when the device is switched on. How might this be tested in a subsequent study?

### 5.2. Reduction in Ulcer Size

Pain is linked with ulcer severity and the stage of healing. As an ulcer heals, pain improves. Whereas an unhealed ulcer can result in worsening of pain, progressing from phase 1 (nociceptive) to phase 2 (neuropathic + nociceptive) and potentially phase 3 (refractory) (reference) [45]. An intervention that accelerates the healing process will likely be beneficial to the pain trajectory. 

Figure 5 shows that pain is positively correlated with ulcer size in this study. Although this correlation does not establish a causal link, it is plausible from the R^2^ value of 0.64 that more than half of the improvement in pain might be attributed to area reduction alone. This must be considered alongside the possibility that improvements in conditions favourable for healing are similar to those conditions favourable for pain reduction. A third possibility is a reverse causation, whereby pain itself impedes healing, for example, by activation of a stress response [46]. It must be noted that the data presented comprises between-subject scatter as well as within-subject scatter, with nine repeated measures being made over an 8-week period for each subject. While the simple Pearson correlation plotted does not account for these mixed effects, the plot remains suggestive that some relationship exists between ulcer size and pain.

### 5.3. Neuromodulation

According to Gate Theory [47], a gate control system modulates sensory input from the skin before it evokes pain perception and response. This explains the effectiveness of TENS (Transdermal Electroneural Stimulation) [48] on pain in numerous pathologies. It is plausible that the NMES device’s stimulation of afferent nerves, directly or indirectly via mechanoreceptors, has a similar gating effect to TENS. However, in contrast to classical TENS devices, which deliver a train of high-frequency pulses, the device used in this study delivers a single charge-balanced pulse only once per second and is targeted at efferent nerves. It is not known whether this would have any substantial neuromodulation effect.

Given the interactions between the three proposed mechanisms for pain reduction, it is difficult to conceive a simple trial methodology to isolate which are effective. 

## 6. Conclusions

Patients with chronic venous ulcers who were given 1 Hz NMES of the common peroneal nerve to activate the leg muscle pump experienced an improvement in their pre-intervention pain trajectory. This mirrors the improvement in the rate of reduction in wound size previously reported in the same cohort when the intervention was introduced. Pain is a common feature of chronic wounds, and its mitigation is likely to have a profound impact on the quality of life of individuals with chronic venous leg ulcers.

## Figures and Tables

**Table 1:** Subject demographics [12].

	SOC		SOC + NMES 12 h Daily		*t*-Test
	Mean	SE	Mean	SE	
Age (years)	67.087	2.074	67.828	2.530	0.828
Height (cm)	175.539	2.689	173.577	2.359	0.585
Weight (Kg)	84.140	5.980	93.397	4.761	0.226
BMI	27.553	1.893	31.048	1.566	0.158
ABPI	1.112	0.017	1.100	0.021	0.666
Wound size (cm^2^)	10.390	1.224	9.961	1.246	0.810
Age of study VLU at enrolment (days)	477.710	104.198	522.801	89.684	0.743
Age of subject at first VLU (years)	54.000	3.461	59.414	2.953	0.237

## Data Availability

The data that support the findings of this study are available from the corresponding author upon reasonable request.
